# Evaluation of Growth Performance, Biochemical Composition, and Polyhydroxyalkanoates Production of Four Cyanobacterial Species Grown in Cheese Whey

**DOI:** 10.3390/microorganisms13051157

**Published:** 2025-05-19

**Authors:** Eirini Sventzouri, Konstantinos Pispas, Georgia G. Kournoutou, Maria Geroulia, Eleni Giakoumatou, Sameh Samir Ali, Michael Kornaros

**Affiliations:** 1 Laboratory of Biochemical Engineering & Environmental Technology (LBEET), Department of Chemical Engineering, University of Patras, 26504 Patras, Greece; eirinisventzouri@gmail.com (E.S.); pispas.msci@gmail.com (K.P.); gkurnutu@upatras.gr (G.G.K.); mariageroulia13@gmail.com (M.G.); eleni.giakoumatou@ac.upatras.gr (E.G.); 2 Botany Department, Faculty of Science, Tanta University, Tanta 31527, Egypt; samh_samir@science.tanta.edu.eg

**Keywords:** cheese whey, cyanobacteria, pigments, phycocyanin, wastewater bioremediation, fatty acid methyl esters, proteins, polyhydroxyalkanoates

## Abstract

Large-scale cultivation of cyanobacteria is often limited by the high cost of synthetic culture medium and the environmental impact of nutrient consumption. Cheese whey, a major agro-industrial waste product, is rich in organic and inorganic nutrients, making it a promising low-cost alternative for microbial growth while addressing waste bioremediation. This study investigates the growth performance and the biochemical composition of four different cyanobacterial species (*Phormidium* sp., *Synechocystis* sp., *Chlorogloeopsis fritschii,* and *Arthrospira platensis*), cultivated in cheese whey (CW). Pretreated CW was used at 20% and 100% *v*/*v* concentrations. All species grew satisfactorily in both concentrations, reaching biomass above 4 g L^−1^ (in 100% *v*/*v* CW) and 2 g L^−1^ (in 20% *v*/*v* CW). The highest μ_max_ value (0.28 ± 0.02 d^−1^) was presented by *Synechocystis* sp. grown in 20% CW. Waste bioremediation of both 20 and 100% *v*/*v* CW demonstrated effective nutrient removal, with COD removal exceeding 50% for most species, while total nitrogen (TN) and total phosphorus (TP) removals reached up to 33% and 32%, respectively. Biochemical composition analysis revealed high carbohydrate and protein content, while lipid content remained below 15% in all cases. Interestingly, *C. fritschii* accumulated 11% *w*/*w* polyhydroxyalkanoates (PHAs) during the last day of cultivation in 20% *v*/*v* CW. These findings highlight the potential of *C. fritschii* as a valuable candidate for integration into bioprocesses aimed at sustainable bioplastic production. Its ability to synthesize PHAs from agro-industrial waste not only enhances the economic viability of the process but also aligns with circular economy principles. This study is a primary step towards establishing a biorefinery concept for the cultivation of cyanobacterial species in cheese whey-based wastewater streams.

## 1. Introduction

Cyanobacteria, also known as blue-green algae, are photosynthetic prokaryotic microorganisms that have attracted significant scientific interest due to their ecological, nutritional, and biotechnological importance. These microorganisms contribute to global carbon and nitrogen cycles by capturing carbon dioxide through photosynthesis and nitrogen fixation, respectively [1]. In recent decades, cyanobacteria have been considered a natural reservoir of diverse active compounds such as pharmaceuticals, pigments, biopolymers, biofertilizers, and high-value nutritional compounds such as proteins, essential fatty acids, and antioxidants [2,3]. Additionally, certain species are of particular interest for their rapid growth rate and ability to adapt to various environmental conditions, making them suitable for the commercial production of high-value products [4].

Polyphenols, carotenoids, polyunsaturated fatty acids, polysaccharides, and phycobiliproteins represent the major antioxidant compounds in cyanobacteria [5]. Cyanobacteria are characterized by a diverse range of fatty acid (FA) profiles. Research into the fatty acid composition has revealed significant levels of long-chain polyunsaturated fatty acids, particularly omega-3 FAs, known for their health benefits. The fatty acid methyl esters (FAMEs) profile is affected by species and culture conditions [6]. Bioactive pigments, besides being a marker for species optimal growth, are widely used in pharmaceutics and neutraceutics, an industry with high profit.

Recent studies indicate the ability of various microorganisms, including bacteria, cyanobacteria, and microalgae, to synthesize bioplastics as intracellular carbon and energy storage compounds, especially under nutrient-limiting conditions [7]. Since plastic-derived pollution has been recognized as a major global concern that demands an urgent solution [8], biodegradable polymers like polyhydroxyalkanoates (PHAs) offer a sustainable alternative. Cyanobacteria, mainly *Synechococcus* sp., *Synechocystis* sp., and *Arthrospira* sp., are among the most promising organisms for PHAs synthesis using various kinds of substrates due to their ability to synthesize these bioplastics from a variety of substrates, including renewable and waste-derived materials [9]. Their photosynthetic capacity and metabolic versatility make them attractive for environmentally friendly bioplastic production systems.

PHAs can be categorized into short-, medium-, and long-chain length PHAs, while the most common PHA, within the majority of microbial cells, is the short-chain length polymer poly-3-hydroxybutyrate, PHB [10]. Poly-3-hydroxybutyrate (PHB) is also the most common bioplastic produced by cyanobacteria.

Large-scale cultivation of cyanobacteria is often limited by the high cost of culture media and the environmental impact of nutrient consumption [11]. Conventional culture systems rely on synthetic media, which increases production costs and may not be sustainable in the long term. Therefore, there is an increasing need to explore alternative, low-cost substrates derived from agro-industrial waste that can simultaneously support microbial growth and contribute to waste valorization [12].

One of the most abundant and underutilized agro-industrial byproducts is cheese whey, a liquid effluent generated during cheese production. Cheese whey (CW) is rich in organic nutrients, particularly lactose, proteins, vitamins, and minerals, making it a potentially valuable substrate for microbial cultivation [13]. However, the disposal of untreated CW poses serious environmental concerns due to its high nutrient concentration, biological oxygen demand (BOD), chemical oxygen demand (COD), and organic and inorganic contents [14,15].

CW has been used in a wide range of applications. These applications either involve the recovery of valuable products (like proteins) or the fermentation of CW with the aim of producing high-added value products (organic acids, lipids, biopolymers) [16]. Anaerobic digestion for methane production from CW has also been used extensively [17]. CW has also been used as an alternative growth medium for the cultivation of microalgae and cyanobacteria, especially for the production of biomass, lipids [18], exopolysaccharides [19], and phycobiliproteins [3]. To the best of our knowledge, there are no other studies that have investigated the production of PHAs by cyanobacteria using CW as substrate.

Valorizing CW through the cultivation of cyanobacteria not only addresses waste management issues but also offers an opportunity to produce valuable biomass for various applications. According to the circular economy principles, growing cyanobacteria in CW can be a viable strategy that might lower production costs while also offering a sustainable way to treat waste from the dairy industry, especially in Europe, which in 2022 was the second largest milk producer globally after India [20].

The objective of this study was to investigate the growth performance of four cyanobacterial species (*Phormidium* sp., *Synechocystis* sp., *Chlorogloeopsis fritschii*, and *Arthrospira platensis*) cultivated in CW-based media using different dilutions of CW obtained from a dairy company. The evaluation of the growth performance of the cyanobacterial species used includes the suitability of CW as a culture medium and the measurement of biomass yield among the selected species. Analysis of the biochemical composition of the produced biomass focuses on cyanobacterial carbohydrates, lipid content (FAMEs profile), proteins, and PHAs. This research aims to contribute to the development of sustainable cyanobacterial cultivation strategies while supporting the valorization of dairy industry waste, improving the economic feasibility and the environmental sustainability of the process.

## 2. Materials and Methods

### 2.1. Cheese Whey

CW was kindly provided by a local dairy company, AVIGAL S.A., located in Patras, Western Greece. Samples were immediately stored at −18 °C prior to use. The physicochemical characteristics of raw CW are presented in Table 1. After CW collection, pretreatment was performed, including filtration through glass fiber filters (Whatman GF/F) with a mean pore diameter of 0.7 μm, to remove solid particles, followed by autoclaving the filtrate at 121 °C for 20 min to prevent cultures contamination, and then centrifugation at 4500 rpm for 5 min (Hermle Z 366, Hermle Labortechnik GmbH, Wehingen, Germany) to remove protein aggregates [21]. The pretreated CW was used as the substrate for the cultivation of selected cyanobacteria.

### 2.2. Cyanobacteria Species and Cultivation Conditions

In total, four cyanobacterial species were used in this study. Specifically, *Chlorogloeopsis fritschii* 1411-1a and *Arthrospira platensis* 21.99 were obtained from the SAG Culture Collection (University of Göttingen), *Synechocystis* sp. PCC 6803 was obtained from the Pasteur Culture Collection of Cyanobacteria, and *Phormidium* sp. was locally isolated from Messolongi, Western Greece [22]. All species were maintained aseptically under continuous illumination of 50 μmol m^−2^ s^−1^. The species *C. fritschii*, *Synechocystis* sp. and *Phormidium* sp. were preserved in BG-11 medium (73816, Sigma-Aldrich, St. Louis, MO, USA) supplemented with a trace metal solution (Mix A5 with Co 92949, Sigma-Aldrich). *A. platensis* was preserved in “Spirulina medium” which contained (g L^−1^) of the following: NaHCO_3_ (13.61), Na_2_CO_3_ (4.03), K_2_HPO_4_ (0.50), NaNO_3_ (2.50), K_2_SO_4_ (1.00), NaCl (1.00), MgSO_4_·7H_2_0 (0.20), CaCl_2_·2H_2_0 (0.04), FeSO_4_·7H_2_0 (0.01), EDTA (Titriplex III, Merck, Rahway, NJ, USA) (0.08), and 0.5% *v*/*v* micronutrient solution containing the following (g L^−1^): ZnSO_4_·7H_2_O (0.001), MnSO_4_·7H_2_O (0.002), H_3_BO_3_ (0.01), Co(NO_3_)_2_·6H_2_O (0.001), Na_2_MoO_4_·2H_2_O (0.001), CuSO_4_·5H_2_O (0.0005·10^−3^), FeSO_4_·7H_2_O (0.7), and EDTA (Titriplex III, Merck) (0.8). For all species, except for *Phormidium* sp., sterilized deionized water was used. In the case of *Phormidium* sp., filtered and sterilized seawater (salinity of 37 ppt) was used in the culture medium.

Prior to experiments, autotrophic pre-cultures of all species were carried out in 500 mL Erlenmeyer flasks with a working volume of 400 mL, cultivated in their proposed medium as described above, under constant temperature at 30 °C, continuous illumination with a photon flux density of 100 μmol m^−2^ s^−1^, measured at the bottom surface of the flasks using a light meter (LI-250A, LI-COR, Lincoln, NE, USA), provided by 6000 K white LED light bulbs placed below the cultures, and filtered atmospheric air at a flow rate of 0.5 L min^−1^. During the late exponential phase, the biomass produced was recovered by centrifugation and used as the inoculum for the following experiments.

**Figure 1:**
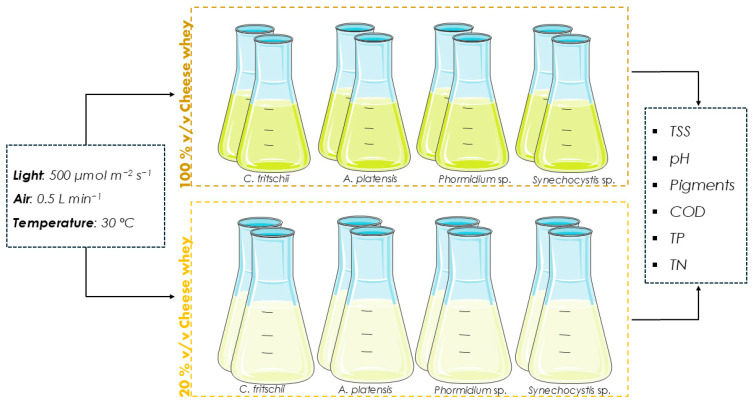
The experimental setup of the present study.

Pretreated CW was used as the substrate for the cultivation of cyanobacteria at two different concentrations, 20% and 100% *v*/*v* (Figure 1). Undiluted CW was fully utilized, in line with a zero-waste strategy and circular economy principles, by maximizing the use of the waste stream and minimizing cultivation costs. Various concentrations of CW were tested to adjust the optical density of the substrate to the desired level, to improve light penetration, and to promote mixotrophic cultivation with rapid pigment production. 20% *v*/*v* was chosen as optimal to initiate pigment production. Cultivation conditions pertaining to temperature and aeration remained the same as those of the autotrophic cultures, while photon flux density was adjusted to 500 μmol m^−2^ s^−1^, considering the substrate’s color. CW was diluted with deionized water, except in the case of *Phormidium* sp., where seawater was used. In the case of undiluted CW, NaCl was added to achieve the desired salinity. For all *A. platensis* cultures, 10 g L^−1^ NaHCO_3_ was added, as a highly alkaline environment has been reported to promote growth [23]. For all other species, pH was adjusted to 7.0 on the first day of the experiment using NaOH 1 N. Due to the aeration and the nature of the substrate, the formation of foam was observed. To prevent biomass loss, 0.1% *v*/*v* antifoaming agent (aqueous emulsion of dimethylpolysiloxane) was added to all cultures. Samples were obtained every 2–3 days to monitor biomass growth and pH. On the last day of each experiment, the bioremediation of wastewater was assessed in terms of COD, TP, and TN removal, while the biochemical composition analysis of the produced biomass was conducted.

### 2.3. Analytical Methods

#### 2.3.1. Biomass Growth

To determine biomass concentration throughout the cultivation period, the Total Suspended Solids (TSS) method was used according to the Standard Methods for the Examination of Water and Wastewater [24]. A sample of a given volume was filtered under vacuum in a weighted filter with a pore diameter of 0.7 μm (Whatman GF/F), so that the cells were retained in the filter. The filter was then rinsed with deionized water, or with 0.5 M ammonium bicarbonate solution (NH_4_HCO_3_) in the case of *Arthrospira platensis* and *Phormidium* sp., and left in an oven at 105 °C until constant weight. The increase in weight of the filter corresponds to the dry weight of the biomass and was calculated based on the following equation. The results were expressed in grams of dry biomass per liter of culture (g DW L^−1^) [24].

(1) $$\mathrm{Biomass}\;\mathrm{concentration}\;\left[g\;DW\cdot L^{-1}\right]=\frac{W_{f}-W_{i}\;\left[g\right]}{V_{s}\;\left[L\right]}\;\;$$

*W*_i_ = Weight of pre-weighed filter, *W*_f_ = Weight of filter after drying, *V*_s_ = Sample volume

The maximum specific growth rate (μ_max_, d^−1^) and maximum biomass productivity (g L^−1^ d^−1^) during the exponential phase of culture growth were determined from the slope of logarithmic plots of dry weight over time.

#### 2.3.2. Cyanobacterial Pigments

In order to extract chlorophylls and total carotenoids, the wet biomass was harvested through centrifugation (4500 rpm, 5 min) and then resuspended in N, N’-dimethylformamide for 20 min at 25 °C. The samples were then centrifuged again, and the absorbance of the supernatant was measured at 480 nm, 646.3 nm, 663.8 nm, and 750 nm. The concentrations of the pigments were calculated according to [25].

For the extraction and the determination of C-phycocyanin, the freeze-thaw method used was based on a previous study [26]. An aliquot of the culture was centrifuged, and the pellet was then resuspended in 1 M Tris-HCl buffer (pH 7) and stored in the freezer (−20 °C). The samples were allowed to thaw at 4 °C for 24 h in a light-proof environment. After thawing, the samples were vortexed, centrifuged, and the supernatant was carefully collected. Finally, the absorbance of the resulting extract was measured using a UV-VIS spectrophotometer (Cary50, Varian, Palo Alto, CA, USA) at four wavelengths (280 nm, 620 nm, 652 nm, and 750 nm). The phycocyanin content was then determined according to the equations provided in an earlier study [27].

#### 2.3.3. CW Remediation

Physicochemical characterization was conducted for the raw CW. Additionally, both organic and inorganic nutrient analyses were performed in the filtrate throughout cyanobacterial cultivation to assess the wastewater’s remediation. Measurements of organic load (via the Chemical Oxygen Demand (COD) assay), total phosphorus (TP), total solids (TS), and volatile solids (VS) were performed following Standard Methods [25]. Total nitrogen (TN) was determined using the persulfate method [24] and a Total Nitrogen Analyzer (TNM-1, Shimadzu, Kyoto, Japan). Total carbohydrates were determined spectrophotometrically using the L-tryptophan-H_2_SO_4_-H_3_BO_3_ method [28].

#### 2.3.4. Biomass Compositional Analysis

At the end of the cultures, the cyanobacterial biomass was collected by centrifugation, washed with deionized water (or 0.5 M NH_4_HCO_3_, as mentioned above), and then lyophilized (LyoQuest, Telstar, Barcelona, Spain). After lyophilization, the intracellular composition of the biomass, including proteins, carbohydrates, lipids, and inorganic matter (ash), was analyzed. Protein content was measured using the semi-micro Kjeldahl method [24], which involved determining total Kjeldahl nitrogen (TKN) and applying a conversion factor of 6.25 to calculate the crude protein content [29]. The carbohydrates in the biomass were quantified using the Dubois total carbohydrate method [30].

The lipid analysis method used required [31,32] the conversion of fatty acids into fatty acid methyl esters (FAMEs) through the process of transesterification. Lyophilized biomass samples were treated with 5 mL of a 10:1:0.3 CH_3_OH:CHCl_3_:H_2_SO_4_ mixture at 90 °C, while shaking constantly. After 2 h, 2 mL of deionized water was added to stop the reaction. The FAMEs were extracted using a 4:1 C_6_H_14_:CHCl_3_ solution, repeated three times to ensure exhaustive extraction. FAMEs analysis was performed using gas chromatography (Agilent Technologies 7890A, Agilent, Santa Clara, CA, USA) with a Flame Ionization Detector (FID). The analysis was carried out using a specific temperature ramp in the oven starting at 40 °C, increased to 195 °C at a rate of 25 °C min^−1^, then from 195 °C to 205 °C at 3 °C min^−1^, and finally ramped from 205 °C to 230 °C at 8 °C min^−1^. Helium was utilized as the carrier gas, maintaining an average velocity of 30.34 cm s^−1^. Both the injector and detector were set at 250 °C. A DB-WAX capillary column (10 m × 0.1 mm × 0.1 μm) was used. A reference standard (FAMQ-005, Accustandard, Inc., New Haven, CT, USA) and an internal standard solution (C17:0, Sigma) were employed.

To determine the PHA content of biomass, an analytical method using gas chromatography was followed, as described by a previous study [33]. The method involves extracting the bioplastic from the cells, followed by the depolymerization and, finally, the transesterification of the monomers. More specifically, around 30 mg of dry biomass was placed in screw-cap glass vials containing 2 mL of chloroform (CHCl_3_). Subsequently, 2 mL of acidified methanol (CH_3_OH) solution (3% *v*/*v* H_2_SO_4_ in methanol) was added. The acidified methanol solution also contained benzoic acid (C_6_H_5_COOH), which serves as an internal standard of the method. The vials were sealed and kept at 95 °C for 4 h, under constant shaking. After four hours, 1 mL of deionized water was added to stop the reaction. The chloroform phase, containing PHAs methyl esters, was filtered through a syringe filter (syringe filter, Nylon/L, 0.22 µm) and injected into the gas chromatograph (GC-FID, Agilent Technologies 7890A). High-purity helium, at a flow rate of 12 mL min^−1^, was used as carrier gas. The injector temperature was set at 175 °C and the detector temperature at 300 °C. A capillary column (DB-FFAP, Santa Clara, CA, USA, 30 m long, 0.25 mm I.D. and 0.25 μm filler) was used.

#### 2.3.5. Statistical Analysis

All experiments were performed in duplicate, and the results are presented as average values along with the calculated standard deviation. One-way ANOVA and Tukey’s test were performed to examine the statistical difference of the calculated values for each species regarding maximum biomass, maximum growth rate, productivity, CW bioremediation, and biomass composition analysis. Significance was established at *p* < 0.05 (null hypothesis). The analysis was performed via Minitab 18 software.

## 3. Results and Discussion

### 3.1. Cyanobacteria Growth

**Figure 2:**
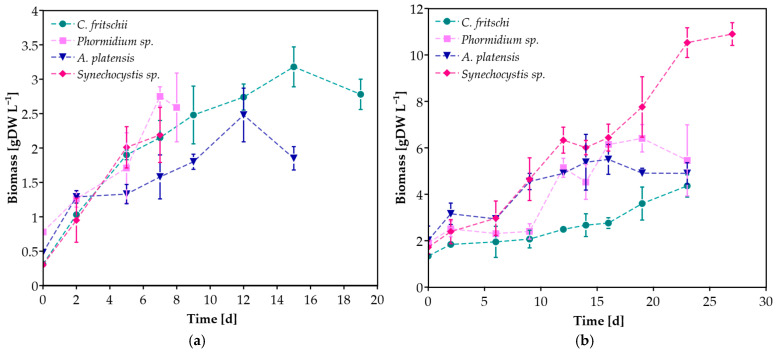
Biomass concentration of cyanobacterial species during cultivation in (**a**) 20%, (**b**) 100% *v*/*v* CW.

As shown in Figure 2, all cyanobacterial species grew successfully in both CW concentrations. For each experimental scenario, maximum biomass concentration, maximum specific growth rate (μ_max_), and productivity were also determined (Table 2). Biomass production seemed to depend mainly on the substrate concentration, indicating the different nutrient requirements of each cyanobacterial species. The highest biomass concentration was observed during the cultivation of *Synechocystis* sp. in undiluted CW, equal to 10.90 ± 0.49 g L^−1^, while it was among the species that exhibited the lowest concentration during the cultivation in 20% CW. On the other hand, *C. fritschii* demonstrated the highest biomass concentration in 20% CW and the lowest at 100% CW among the other species. In the case of undiluted CW, the biomass of all cyanobacteria cultures reached a concentration greater than 4 g L^−1^. The highest μ_max_ value (0.28 ± 0.02 d^−1^) was presented by *Synechocystis* sp. grown in 20% CW, while μ_max_ for all species grown in 100% CW was lower without statistical significance, ranging between 0.07 and 0.11 d^−1^. Casa et al. [34] also reported higher values of μ_max_ when increasing the dilution of ricotta CW with BG-11 during *C. vulgaris* cultivation. However, high productivity values were reported for all species and cultivation conditions (Table 2). Significantly lower productivities than those reported in this study were observed when *C. vulgaris*, *T. obliquus*, and *D. tertiolecta* were cultivated in CW with 5 g L^−1^ glucose, corresponding to 0.124, 0.116, and 0.071 g L^−1^ d^−1^, respectively [35]. According to the literature, the highest biomass concentration (equal to 8 g L^−1^) using CW as a substrate was reported in the study of Bonnet et al. [36] during the cultivation of the locally isolated microalgae *Desmodesmus* sp. L2B Bolt in CW permeate. However, this biomass concentration is lower than the achieved 10.90 g L^−1^ of *Synechocystis* sp. cultivated in undiluted CW in this study. High value was also reported during the cultivation of *S. obliquus* at 40% CW diluted with Bold’s basal medium, reaching 2.6 g L^−1^ [18]. Among the cyanobacterial species used in this study, *A. platensis* has been studied for its cultivation in CW, though in lower concentrations (0.8–10% *v*/*v*) [3,37,38].

Overall, the biomass concentration of all species in both concentrations ranged between 2.19 ± 0.40 and 10.90 ± 0.49 g L^−1^, much higher than the reported values of several microalgae and cyanobacteria autotrophic cultivation in their proposed synthetic medium, indicating that CW benefits cyanobacteria growth and can be used as an alternative substrate reducing the cultivation cost [39]. Pandey et al. [40] proposed an integrated process for biofuel production from microalga *Chlorella pyrenoidosa*, utilizing CW wastewater for biomass accumulation instead of a synthetic medium, with an estimated annual profit of US$9.59 million.

**Figure 3:**
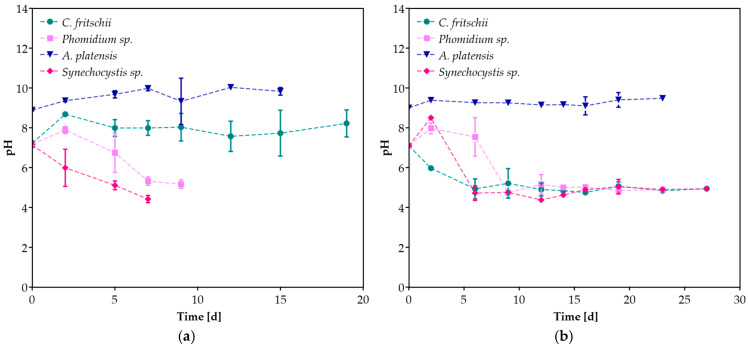
The pH variation of cyanobacterial species during cultivation in (**a**) 20%, (**b**) 100% *v*/*v* CW.

During the cultivation period, pH was also monitored (Figure 3). pH values for *A. platensis* remained at the desired levels with the addition of NaHCO_3_ [38]. While the initial pH was set at 7.0 before inoculation, a decrease in its value was observed in all cases except for the cultivation of *C. fritschii* at 20% *v*/*v* CW. In the study of Salah et al. [41], a decrease in pH value was also reported during the cultivation of *Desmodesmus* sp. At different CW concentrations. The decrease in pH during mixotrophic cultivation in CW can be attributed to the conversion of soluble organic monomers into organic acids via acidogenesis and acetogenesis [42]. The study of Youssef et al. [43] proved that pH and the interaction between pH and whey concentration had a significant effect on the biomass production of *T. obliquus*.

### 3.2. Pigments

**Figure 4:**
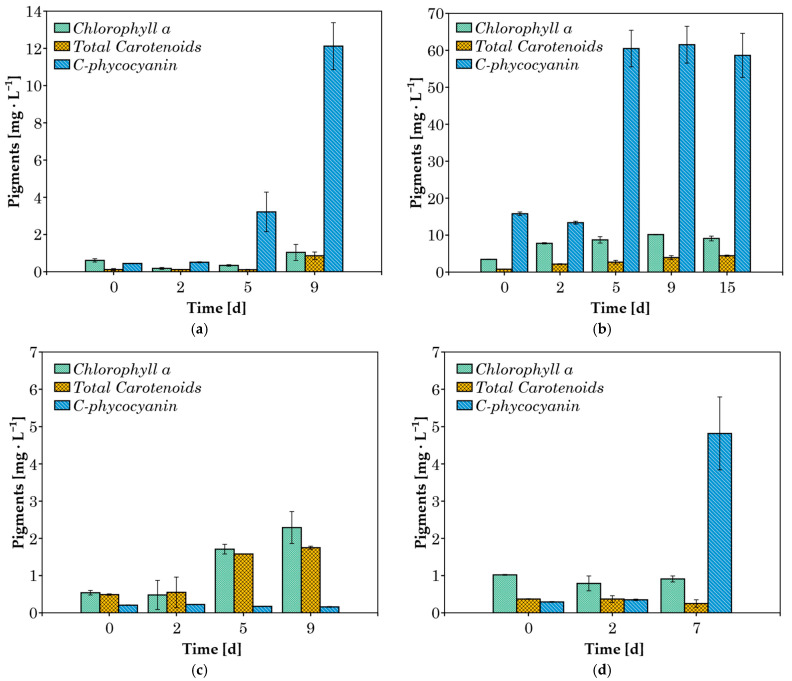
Concentration of pigments during cultivation in 20% *v*/*v* CW for (**a**) *C. fritschii*; (**b**) *A. platensis*; (**c**) *Phormidium* sp.; (**d**) *Synechocystis* sp.

The pigment concentrations (chlorophyll a, total carotenoids, and C-phycocyanin) were measured in cultures grown in 20% *v*/*v* CW (Figure 4). When cyanobacteria were cultured in 100% *v*/*v* CW substrate, both lipophilic pigments and phycocyanin were not detected in significant amounts after the second day of cultivation, likely due to the heterotrophic growth of the cyanobacteria [44]. Therefore, these results are not presented here. The absence of pigment production could enhance scalability by redirecting metabolic energy toward biomass accumulation, simplifying downstream processing, and improving culture stability under heterotrophic conditions. The absence of pigments corresponds with the major advantages of heterotrophic cultivation, such as higher cell densities, better process control, and greater economic efficiency [45].

In *A. platensis* cultures grown in 20% *v*/*v* CW, chlorophyll a and carotenoid concentrations increased throughout the exponential growth phase, up to day 9 (Figure 4b). This increase in chlorophyll a is attributed to the activation of photoautotrophic metabolism, as pigments serve as indicators of this process [46]. Since CW is a turbid liquid, the growth of cyanobacteria in this medium can lead to low incoming light intensity in the culture, resulting in growth inhibition and negative effects on photosystem II [47]. In response to this obstacle, the cells overproduce accessory light-harvesting pigments (phycobiliproteins), which can also absorb light at wavelengths where chlorophylls are unable to [48]. This pattern of increasing C-phycocyanin concentration over the course of several days was observed in three of the four species examined. *A. platensis* achieved a maximum C-phycocyanin concentration of around 61 mg L^−1^ after the 5th day of cultivation (Figure 4b). *C. fritschii* and *Synechocystis* sp. cultures achieved maximum C-phycocyanin contents of 12.1 and 4.8 mg L^−1^, respectively. In contrast, *Phormidium* sp., which is known to be an efficient strain in phycocyanin production [22,49], did not produce any significant amount of the pigment (Figure 4c) under these conditions. The lower pigment concentrations observed during the cultivation of *Phormidium* sp., *C. fritschii,* and *Synechocystis* sp. may be attributed to heterotrophic metabolism.

### 3.3. CW Bioremediation

An evaluation of CW bioremediation was conducted, with the assessment parameters comprising COD, TP, and TN. The removal values were determined on the final day of each cultivation (Table 3). The pretreatment of CW did not significantly affect the physicochemical characteristics of the substrate, with the initial concentrations of COD, TN, and TP (in g L^−1^) being 54.1 ± 1.4, 1.0 ± 0.0, and 0.3 ± 0.0, respectively. High COD removal (above 50%) was observed for most of the species, while the lowest value was reported for *A. platensis* at 20% *v*/*v* CW (11%). However, COD removal during cultivation in diluted CW was not significantly different compared to undiluted substrate, indicating the mixotrophic growth of cyanobacteria. In the study conducted by Hemalatha et al. [50], it was reported that 90% of the COD was removed during the cultivation of a mixed microalgal culture in dairy wastewater, with an initial COD concentration of 1.8 g L^−1^. Efficient TP and TN removals were also observed. During the cultivation of *Chlorella* sp. in undiluted secondary CW, TN and TP removals were 33% and 32%, respectively [51]. However, higher removal values were reported in the study of Sánchez-Zurano et al. [52]. In none of the cases studied, culture’s nutrients were completely consumed, indicating that they were not the limiting factor. Cyanobacterial growth was probably restricted due to limited light penetration, while the remaining organic matter was difficult to further biodegrade.

### 3.4. Biomass Composition

Table 4 presents the biochemical composition of the four different cyanobacterial species under two different culture conditions: 20% and 100% *v*/*v* CW. The parameters measured include ash content, carbohydrates, proteins, lipids, and PHAs, all reported in percentage on a dry basis. Inorganic components of the biomass, determined as % ash, seemed to vary among the species, with *A. platensis* and *Phormidium* sp. showing quite higher values, compared to *C. fritschii* and *Synechocystis* sp. The high inorganic content in *A. platensis* may be due to its greater intracellular accumulation of phosphorus, which can be up to four times higher than in other species [53]. For *C. fritschii,* the inorganic content decreased significantly in the case of 100% CW, whereas for *Synechocystis* sp., it remained relatively stable. Koutra et al. [54] also reported that the addition of CW in *Chlorella vulgaris* cultures led to an increase in the ash content of the microalgal biomass.

Carbohydrate levels did not significantly vary. Sanchez-Zurano et al. [52] observed a progressive increase in the carbohydrate content of *Chlorella vulgaris* biomass with the addition of varying concentrations of CW (2.5, 5, 10%). From Table 4, it can be concluded that proteins are the most abundant intracellular component for most species. Protein content is particularly high in *Synechocystis* sp., especially in 20% CW (47.9%). The protein content in the biomass varied significantly depending on the microbial species, ranging between 30% and 55% of dry weight [55]. In our case, protein levels tended to increase using 100% CW, except in *Synechocystis* sp., where a slight decrease was observed.

**Figure 5:**
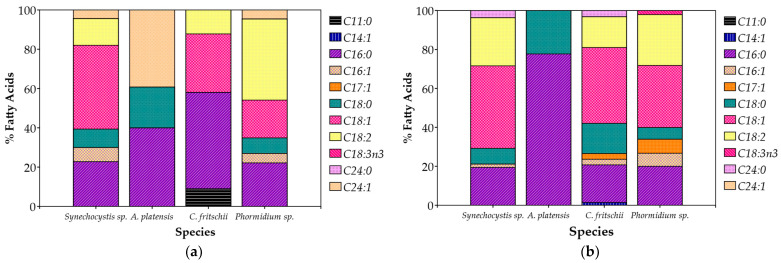
Fatty acid profile (%) of total fatty acids of cyanobacterial biomass cultivated in (**a**) 20% *v*/*v*; (**b**) 100% CW.

*Synechocystis* sp. and *C. fritschii* accumulated more lipids in 100% CW, while *A. platensis* and *Phormidium* sp. maintained stable lipid levels for both of the CW percentages (Table 4). The composition of fatty acids (FAs) of the cultivated cyanobacteria showed distinct differences (Figure 5). In summary, unsaturated fatty acids were the majority, contributing to 50–70% of total FAs, while saturated fatty acids were present in 30–50%. Palmitic acid (C16:0), oleic acid (C18:1), and linoleic acid (C18:2) were the most abundant fatty acids, common for all species. Palmitic acid made up 20% to 50% of the overall fatty acids, and oleic and linoleic acids combined contributed just under 50%. In addition to these dominant fatty acids, smaller amounts of monounsaturated fatty acids like palmitoleic acid (C16:1) were detected, typically making up less than 10% of the total. Stearic acid (C18:0) was also present, but generally in lower concentrations, usually between 10% and 20%. Myristoleic acid (C14:1) was only detected in *C. fritschii* cultivated in 20% CW, though in very small amounts (Table 4). Interestingly, α-Linolenic acid (C18:3n3), an omega-3 essential fatty acid, was found only in *Phormidium* sp. and only in the case of 20% CW.

The ability of all cyanobacterial species to accumulate PHAs was also investigated in all tested culture conditions. As shown in Table 4, the PHAs synthesizing capacity differed significantly among cyanobacterial strains depending on genetic background and other environmental and metabolic factors, as well as cultivation factors [56]. Notably, *C. fritschii* exhibited the highest PHAs content, reaching 10.7% of dry weight after cultivation in 20% diluted CW, while PHAs accumulation dramatically decreased, almost to zero, after cultivation in 100% CW. This strong dependence on substrate concentration suggests that moderate nutrient stress, as induced by diluting CW, favors PHAs biosynthesis in *C. fritschii*. In contrast, *A. platensis*, *Phormidium* sp., and *Synechocystis* sp. showed consistently low PHAs levels in both CW concentrations, with maximum values not exceeding 2.8% in *Synechocystis* sp. at 20% diluted CW. For all three species studied, PHAs production appeared relatively insensitive to variations in culture conditions and consequently nutrient levels. This is possibly due to species-specific metabolic priorities favoring protein or carbohydrate accumulation over carbon storage polymers. Our findings are particularly noteworthy, as there are scarce studies on PHAs production during the cultivation of photosynthetic microorganisms in CW.

However, these results are similar to those in the study by Zhang et al. [57], in which *C. fritschii* was cultivated in BG-11 with the addition of acetate, which has been reported to enhance PHAs accumulation. In general, PHAs production from CW can be achieved through the direct conversion of lactose to PHAs, a process capable of a limited number of bacteria, by the conversion of glucose/galactose and lactic acid [15], which may also explain why pH values of *C. fritschii* did not significantly decrease compared to other species (Figure 3a).

## 4. Conclusions

Overall, this study investigated the growth performance of *Chlorogloeopsis fritschii*, *Arhtrospira platenis*, *Phormidium* sp., and *Synechocystis* sp. in different CW concentrations. All cyanobacterial species successfully grew in CW, achieving high biomass concentrations in both cases, with a higher maximum growth rate being reported in the case of diluted CW. Cultivation also resulted in sufficient bioremediation of the wastewater, with chemical oxygen demand removal exceeding 50%, while nutrients removal was also high. Cyanobacterial biomass was characterized by high carbohydrate and protein content, while the lipid content remained at a lower level. It is worth mentioning that *C. fritschii* accumulated high bioplastic (PHAs) content, 11% *w*/*w*, during its cultivation in diluted CW. The production of PHAs by *C. fritschii* represents a significant advancement in the field of sustainable bioplastics. Its ability to synthesize PHAs using agro-industrial waste such as CW offers a dual benefit: valorization of waste streams and production of high-value biopolymers. The observed accumulation of 11% *w*/*w* PHAs under nutrient-rich yet diluted conditions highlights the organism’s metabolic flexibility and its suitability for industrial-scale applications. Moreover, this biological route to bioplastic production aligns with circular economy models, contributing to reduced environmental impact, improved waste management, and the development of sustainable materials. Therefore, *C. fritschii* emerges as a promising candidate for future bioprocesses integrating wastewater treatment and biopolymer synthesis. While CW represents a sustainable and cost-effective substrate for the cultivation of cyanobacteria and the production of value-added components, challenges remain for the upscaling process. Variability in the CW composition, high risk of culture contamination, and the need for extensive pretreatment are barriers that need to be addressed to achieve widespread application.

Although the results of this study on biomass production and CW remediation are encouraging, little is known about the mechanisms responsible for cyanobacterial adaptability, nutrient assimilation, and bioplastic accumulation. Further investigation at the transcriptomic and metabolomic level will unravel regulatory mechanisms and metabolic pathways activated during growth in CW-based media. In this context, particularly in the case of *C. fritschii,* which showed the ability to accumulate PHAs, further studies for the optimization of culture conditions remain to be performed to achieve optimal productivity. Understanding the metabolic fluxes related to cell growth and maintenance will support the development of tailored cultivation strategies within a systems biology framework. High-throughput technologies are a valuable tool for bioprocess optimization methodologies, along with scale-up simulation, which is the future perspective of studying the growth of cyanobacterial species in CW.

In conclusion, CW appears to be an alternative substrate for cyanobacterial growth and the production of value-added compounds, achieving simultaneously wastewater bioremediation, establishing a cost-effective and environmentally friendly process.

## Figures and Tables

**Table 1:** Physicochemical characteristics of raw CW. Data are presented as means ± SD (*n* = 3).

Parameters	Mean Value ± SD
pH	5.3 ± 0.1
Total suspended solids (TSS) [g L^−1^]	6.5 ± 0.7
Volatile suspended solids (VSS) [g L^−1^]	0.6 ± 0.1
Total solids (TS) [g L^−1^]	56.5 ± 0.4
Volatile solids (VS) [g L^−1^]	51.9 ± 1.3
Chemical oxygen demand (COD) [g L^−1^]	65.0 ± 5.0
Carbohydrates * [g L^−1^]	43.3 ± 1.2
Total nitrogen (TN) [g L^−1^]	0.9 ± 0.1
Total phosphorus (TP) [g L^−1^]	0.4 ± 0.0

* In glucose equivalent.

**Table 2:** Maximum biomass concentration, maximum growth rate (μ_max_), and exponential growth phase productivity of cyanobacterial species during cultivation in 20% and 100% *v*/*v* CW.

	*C. fritschii*		*A. platensis*		*Phormidium* sp.		*Synechocystis* sp.	
	20% CW	100% CW	20% CW	100% CW	20% CW	100% CW	20% CW	100% CW
Maximum Biomass [g L^−1^]	3.18 ± 0.29 ^d,e^	4.36 ± 0.48 ^c,d^	2.48 ± 0.39 ^e^	4.90 ± 0.46 ^b,c^	2.75 ± 0.14 ^d,e^	6.41 ± 0.59 ^b^	2.19 ± 0.40 ^e^	10.90 ± 0.49 ^a^
μ_max_ [d^−1^]	0.21 ± 0.03 ^b^	0.08 ± 0.01^c^	0.11 ± 0.01 ^c^	0.08 ± 0.01 ^c^	0.18 ± 0.01 ^b^	0.11 ± 0.00 ^c^	0.28 ± 0.02 ^a^	0.08 ± 0.01 ^c^
Productivity [g L^−1^ d^−1^]	0.24 ± 0.05 ^b,c^	0.38 ± 0.02 ^a,b^	0.17 ± 0.03 ^c^	0.36 ± 0.08 ^a,b,c^	0.29 ± 0.01 ^a,b,c^	0.48 ± 0.02 ^a^	0.27 ± 0.10 ^b,c^	0.38 ± 0.02 ^a,b^

Data in the same row that do not share a common lowercase letter are significantly different based on the Tukey test (*p* < 0.05).

**Table 3:** CW bioremediation in terms of chemical oxygen demand, total phosphorus, and total nitrogen of cyanobacterial species during cultivation in 20% and 100% *v*/*v* CW.

	*C. fritschii*		*A. platensis*		*Phormidium* sp.		*Synechocystis* sp.	
	20% CW	100% CW	20% CW	100% CW	20% CW	100% CW	20% CW	100% CW
COD_removal_ [%]	51.4 ± 0.5 ^a^	52.3 ± 11.5 ^a^	11.1 ± 1.6 ^c^	17.1 ± 3.0 ^b,c^	47.9 ± 7.9 ^a^	42.9 ± 11.2 ^a,b^	49.4 ± 5.3 ^a^	57.9 ± 3.9 ^a^
TP _removal_ [%]	32.8 ± 5.8 ^a,b,c^	41.2 ± 10.1 ^a,b^	41.6 ± 1.7 ^a,b^	11.6 ± 2.2 ^c^	27.5 ± 6.1 ^a,b,c^	37.1 ± 5.8 ^a,b^	19.1 ± 7.7 ^b,c^	44.6 ± 6.6 ^a^
TN _removal_ [%]	53.1 ± 2.0 ^a,b^	30.4 ± 4.6 ^b,c^	55.9 ± 2.5 ^a^	40.0 ± 10.2 ^a,b,c^	41.2 ± 6.6 ^a,b,c^	48.3 ± 5.1 ^a,b^	18.4 ± 5.2 ^c^	59.0 ± 9.3 ^a^

Data in the same row that do not share a common lowercase letter are significantly different based on the Tukey test (*p* < 0.05).

**Table 4:** Biomass composition (%), on dry basis, of cyanobacterial species during cultivation in 20% and 100% *v*/*v* CW.

	*C. fritschii*		*A. platensis*		*Phormidium* sp.		*Synechocystis* sp.	
	20% CW	100% CW	20% CW	100% CW	20% CW	100% CW	20% CW	100% CW
Carbohydrates [%]	38.1 ± 2.4 ^a^	28.7 ± 2.7 ^a^	24.7 ± 10.1 ^a^	16.7 ± 5.7 ^a^	19.6 ± 6.7 ^a^	29.4 ± 1.9 ^a^	35.9 ± 4.8 ^a^	34.2 ± 5.3 ^a^
Proteins [%]	22.2 ± 3.4 ^b^	41.6 ± 5.3 ^a,b^	34.0 ± 0.1 ^a,b^	38.4 ± 1.0 ^a,b^	45.5 ± 2.9 ^a^	42.4 ± 1.7 ^a,b^	47.9 ± 13.3 ^a^	39.3 ± 1.8 ^a,b^
Lipids [%]	1.0 ± 0.1 ^b^	16.8 ± 1.2 ^a^	10.8 ± 3.2 ^a,b^	1.7 ± 0.7 ^b^	3.8 ± 1.7 ^b^	7.4 ± 2.7 ^a,b^	4.2 ± 2.8 ^a,b^	11.1 ± 7.5 ^a,b^
PHAs [%]	10.7 ± 2.1 ^a^	0.6 ± 0.0 ^b^	0.6 ± 0.0 ^b^	0.9 ± 0.4 ^b^	0.4 ± 0.1 ^b^	1.4 ± 0.1 ^b^	2.8 ± 1.4 ^b^	0.5 ± 0.0 ^b^
Ash [%]	8.5 ± 1.9 ^b,c^	4.7 ± 0.8 ^c^	22.5 ± 2.4 ^a,b,c^	28.1 ± 5.3 ^a^	26.0 ± 8.5 ^a,b^	13.3 ± 5.9 ^a,b,c^	8.4 ± 2.3 ^b,c^	9.0 ± 4.2 ^b,c^

Data in the same row that do not share a common lowercase letter are significantly different based on the Tukey test (*p* < 0.05).

## Data Availability

The raw data supporting the conclusions of this article will be made available by the authors on request.

## Abbreviations

The following abbreviations are used in this manuscript:

BOD	Biological oxygen demand
COD	Chemical oxygen demand
CW	Cheese whey
DW	Dry weight
FAMEs	Fatty acid methyl ester
FAs	Fatty acids
PHAs	Polyhydroxyalkanoates
PHB	Polyhydroxybutyrate
TKN	Total Kjeldahl nitrogen
TN	Total nitrogen
TP	Total phosphorus
TS	Total solids
TSS	Total suspended solids
VS	Volatile solids
